# Supplementary material for: Movement Protein Mediates Systemic Necrosis in Tomato Plants with Infection of Tomato Mosaic Virus
Source: Viruses. 2023 Jan 4;15(1):157. doi: 10.3390/v15010157 (PMC9861833; doi:10.3390/v15010157)
Supplement: Supplementary file 1 [file viruses-15-00157-s001.zip › viruses-2110018-supplementary.pdf]

Table S1. Primers used in vector construction and oligonucleotides for RNA blot

Primer name	Primer sequence(5'- 3')	Brief description
P1	GTATTTTTACAACAATTACCAACAACAAC	pCB301-N5 pCB301-S1
P2	ATCTCTAATGATACTAACTAAAGGATCCATC	
P3	TGATGGATCCTTTAGTTAGTATCATTAGAGA	
P4	TGGGCCCCwACCGGGGGT	
P5	AATTCGTTAGGTAACCAGTTTCAAAC	pCB301- -S1$^{N5\text{-}cp}$ or N5$^{S1\text{-}cp}$
P6	TTAAGATGCAGGTGCAGAGGTC	
P7	gtctgggttggtctgGACCTCTGCACCTGCATCTTAAA	
P8	CGATCGGGGAAATTCGAGC	
P9	ATGGCTCTAGTTGTTAAAGGTAAGGTAA	pCB301-Δcp-*gfp*
P10	TGACAAAAACACAAATTGCGATGGAGAAGTGATTGAGTAAGACcTATTTAATACGAATC	
P11	attcgtattaaataggtACGCGTcccgggATGCATAGGTGCyGAAATATAAAGTTTG	
P8	CGATCGGGGAAATTCGAGC	
P12	CGacgcgtATGGTGAGCAAGGGCGAGGAG	
P13	TCCcccgggTTACTTGTACAGCTCGTCCATGC	
P14	TCCcccgGGTAGTCAAGATGCATAATAAATAACG	pCB301-N5-*gfp* pCB301-S1-*gfp*
P15	TGGGCCGCTACCGGCGGT	
P16	ccggtagcggcccaGGGTCGGCATGGCATCTC	
P8	CGATCGGGGAAATTCGAGC	
P21	CGggatccATGGCTCTAGTTGTTAAAGGTAAGGTAA	pCB301-N5-mp and S1-mp
P22	TCCcccgggTTAATACGAATCAGAATCCGCGAC	
P24	TCTAGTTGTTAAAGGTAAGGTAAATATTAATG	MP mutants of S1-*gfp*, N5-*gfp,* S1
P25	GCAGACACTCACACCACCAC	
P26	GTGGTGTGAGTGTCTGCWTGGTTGACAAGAGAATGGAAAGAG	
P27	TATTATTTTTATAAACAATACACACAGACACA	
P28	ATTGTTTATAAAAATAATAYAAAATTGGGTTTGAGGGAGAAAGT	
P29	AAAACTTTTTGGTTTAGGCCTTCC	
P30	CCTAAACCAAAAAGTTTTKATGAAGTTGAAAAAGAGTTTGATAATTTG	
P31	TGTGTTTGAAACTGGTTACCTAACG	
P32	CCGCGCGCTCCAAGACACTACCCTTCGATTTAA-DIG	ToMV RNAs
P33	GTGTGGACAGGTAATGGTTGTCTGGTAAAAGGACAGGGC-DIG	GFP mRNA
P34	TTGCGTGTCATCCTTGCGCAGG-DIG	U6 RNA

P35-37	TCAAGAGCGCCATGCCTGAGGGATACGTGCAGGAGA-DIG, AGGACCATCTTCTTCAAGGACGACGGGAACTACAAGACACG-DIG and GTGCTGAAGTCAAGTTTGAGGGAGACACCCTCGTCAACAG-DIG	*GFP*-siRNA
