# Supplementary material for: Movement Protein Mediates Systemic Necrosis in Tomato Plants with Infection of Tomato Mosaic Virus
Source: Viruses. 2023 Jan 4;15(1):157. doi: 10.3390/v15010157 (PMC9861833; doi:10.3390/v15010157)
Supplement: Supplementary file 1 [file viruses-15-00157-s001.zip › viruses-2110018-supplementary.pptx]

## Slide 1
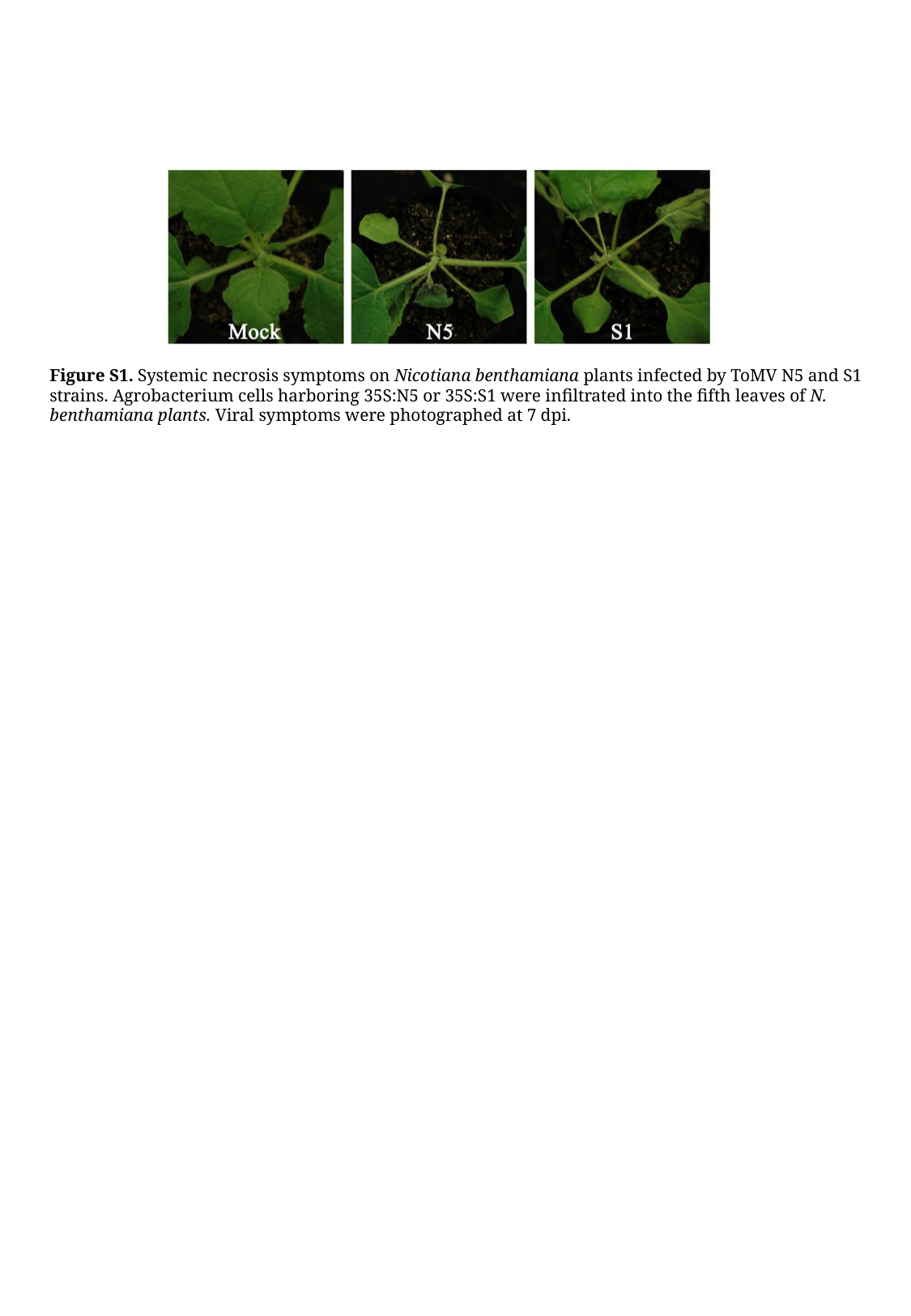

Figure S1. Systemic necrosis symptoms on Nicotiana benthamiana plants infected by ToMV N5 and S1 strains. Agrobacterium cells harboring 35S:N5 or 35S:S1 were infiltrated into the fifth leaves of N. benthamiana plants. Viral symptoms were photographed at 7 dpi.

## Slide 2
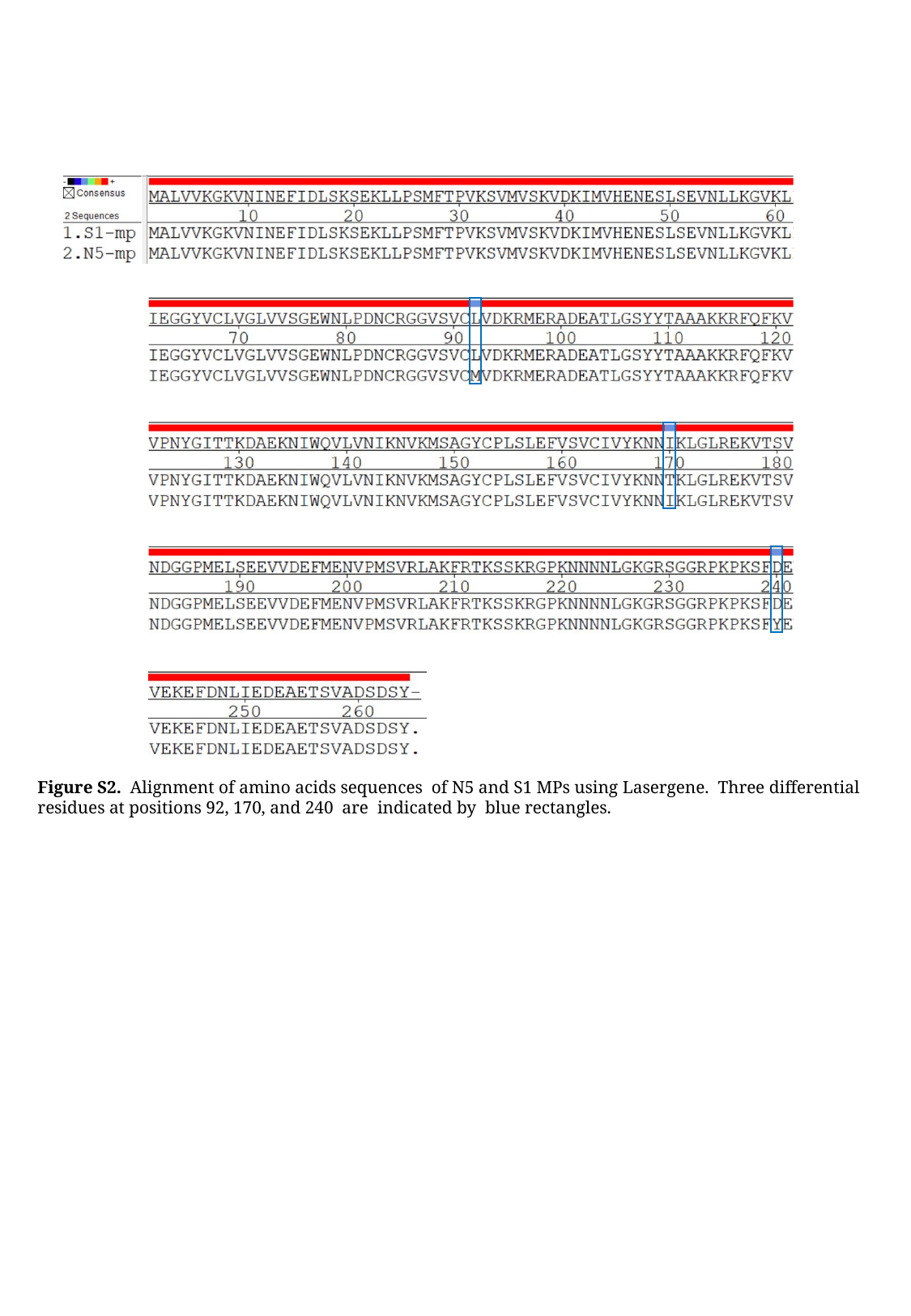

Figure S2. Alignment of amino acids sequences of N5 and S1 MPs using Lasergene. Three differential residues at positions 92, 170, and 240 are indicated by blue rectangles.
